# *iScience*: A groundbreaking journey of innovation and knowledge

**DOI:** 10.1016/j.isci.2023.107442

**Published:** 2023-08-17

**Authors:** Simona Fiorani

**Affiliations:** Executive Editor, *iScience*

Dear Reader,

Welcome to *iScience*.

I am writing this letter to reflect on the successes that have characterized the first 5 years of this amazing, fun, and different journal, and to share the direction that we intend to pursue in the future.

When we started this journey, we promised to our community that we would strive to make this journal “the exception in many ways.” I believe we have succeeded in our mission, and I hope you will agree with me.

The idea was to create a journal that was not out there yet. A product that would be inclusive of many areas of research, with the mission of making science immediately available and foster interdisciplinary collaborations.

To achieve this goal, we set out new avenues to share stories around interdisciplinary collaborations, interdisciplinary research programs and approaches to interdisciplinary research in different institutes around the globe with the mission of stimulating discussion, and share victories and failures, to accelerate the normalization of this approach to scientific questions. We called these types of publications Backstories.

But, we did not stop there. We started a program of special issues with the intent of recruiting and publishing truly interdisciplinary research that would bring together scientific communities that may not naturally interact. At *iScience,* we strongly believe that this is what is needed to answer the great scientific questions society faces and to bring to life impactful solutions, faster.

As the journal grew in readership, we evolved the published content from physical sciences (Best of Physical Science) and life sciences (read the latest collections: Plant Biology and Application of Cardiomyocytes), to include earth (read the latest collection: Geoscience & Sustainability) and health (read the latest collection: Translational Research) sciences and bring into discussion interdisciplinary collaborations that will result in better answers to critical sustainability and health challenges. We have already published content in the social sciences to some extent (Multidisciplinary Highlights in the Social Sciences), and we will be expanding to cover more topics in this area, in the near future. This is extremely important to the identity of the journal as social sciences cut across many of the fields we are publishing.

Our editorial structure has also evolved with time, and I feel that we have found a great solution to serve at best our broad readership, in embracing a multifaceted model. The journal is run by professional editors (Scientific Editors) that dedicate their time to assessing manuscripts and managing the peer review, on top of commissioning great content for our special issues and Backstories. The professional editors are experts in a limited number of areas of research and therefore are assisted by our Consulting Editors. These editors are academics that help manage the peer review and the commissioning of content in specific areas of research where either we are missing expertise in our professional editorial team or we are actively expanding our footprint. Their contribution is essential to keep us engaged and connected with the many communities we serve.

Finally, our Advisory Board helps us with more strategic decisions, advising us on the direction of research in their specific fields and advocating for interdisciplinary research in their communities.

Our Scientific Editors, Consulting Editors, and Advisory Board members are from various backgrounds and are located all over the world in support of our pledge for diversity and inclusion.

In the future, we aim to continue to publish impactful research and facilitate sharing of information others can build upon, in the fastest and fairest way possible. As the journal expanded tremendously, this has been challenging, at times, but all editors and I are committed to deliver on this promise. We thank both authors for entrusting us with their research and reviewers for supporting us by delivering timely and thoughtful comments for authors.

As *iScience* continues to establish itself as a strong voice in the scientific community, we are looking forward to continuing to engage with scientists across all career stages to break the barriers across disciplines and facilitate collaborations in the name of what we love the most: science.

